# 579. Clinical and Drug Resistance Characteristics of *Providencia spp.* Infections

**DOI:** 10.1093/ofid/ofad500.648

**Published:** 2023-11-27

**Authors:** Pramodini Kale-Pradhan, Lauren Lim, Heather Graskewicz, Meenal Malviya, M E R E D I T H M COYLE, Christopher Giuliano, Leonard B Johnson

**Affiliations:** Wayne State University Eugene Applebaum College of Pharmacy and Health Sciences, Detroit, Michigan; Wayne State University Eugene Applebaum College of Pharmacy and Health Sciences, Detroit, Michigan; Wayne State University Eugene Applebaum College of Pharmacy and Health Sciences, Detroit, Michigan; Ascension St. John Hospital, Detroit, Michigan; aSCENSION ST JOHN, GROSS POINTE WOODS, Michigan; Wayne State University Eugene Applebaum College of Pharmacy and Health Sciences, Detroit, Michigan; Ascension St. John Hospital, Detroit, Michigan

## Abstract

**Background:**

*Providencia* is a gram-negative bacillus that colonizes the urinary tract and is often resistant to many antimicrobials. This study aimed to evaluate resistance patterns of *Providencia spp* (*P. spp) and* clinical outcomes due to the paucity of topical data.

**Methods:**

A multi-center, descriptive, retrospective chart review of adult patients with *P. spp*. infections was conducted from January 1, 2020 to May 31, 2022. The primary outcome was to describe the drug resistance patterns of *P. spp*. isolates. The study’s secondary outcome was to evaluate the clinical outcomes of patients with *P. spp* infections.

**Results:**

Of 312 patients screened, 243 were excluded primarily for polymicrobial infections. The mean age was 70 years and 39 (56.5%) were males. Of the 69 included cases, 46 (66.7%) were *P. stuartii,* 21 (30.4%) *P. rettgerri*, and 2 (2.9%) *P. alcalifaciens.* The most common infections were bacteremia 38 (55.1%), with urinary and wound being the most frequent bacteremia source; followed by 28 (40.6%) urinary tract and 3 (4.3%) wound infections. 45 patients (65.2%) had urinary catheters. Primary antibiotics used for treatment consisted of ceftriaxone, 25 (36.2%), cefepime, 20 (29%), and meropenem, 10 (14.5%). Five of 69 (7.2%) cases were multidrug resistant and required meropenem. Nineteen patients (27.1%) died during their admission but none related to *Providencia* infections. Ten of the 69 patients (14.5%) were readmitted within 30 days for reasons unrelated to progression or recurrence of *Providencia* infections.

**Conclusion:**

*Providencia* bacteremia was the most common infection. Third generation cephalosporins remain an appropriate choice of antibiotics for *P. spp*. However, *P. stuartii* was the only species with multidrug resistance. Future studies should be carried out to confirm this observation.

**Disclosures:**

**All Authors**: No reported disclosures

